# Analysis of the bacterial communities associated with two ant–plant symbioses

**DOI:** 10.1002/mbo3.73

**Published:** 2013-02-17

**Authors:** Ryan F Seipke, Jörg Barke, Darren Heavens, Douglas W Yu, Matthew I Hutchings

**Affiliations:** 1 School of Biological Sciences, University of East AngliaNorwich Research Park, Norwich, NR4 7TJ, United Kingdom; 2 The Genome Analysis CentreNorwich Research Park, Norwich, NR4 7UH, United Kingdom

**Keywords:** 16S pyrosequencing, *Allomerus*, fungus-growing ants, microbiome, *Tetraponera*

## Abstract

Insect fungiculture is practiced by ants, termites, beetles, and gall midges and it has been suggested to be widespread among plant–ants. Some of the insects engaged in fungiculture, including attine ants and bark beetles, are known to use symbiotic antibiotic-producing actinobacteria to protect themselves and their fungal cultivars against infection. In this study, we analyze the bacterial communities on the cuticles of the plant–ant genera *Allomerus* and *Tetraponera* using deep sequencing of 16S rRNA. *Allomerus* ants cultivate fungus as a building material to strengthen traps for prey, while *Tetraponera* ants cultivate fungus as a food source. We report that *Allomerus* and *Tetraponera* microbiomes contain >75% Proteobacteria and remarkably the bacterial phyla that dominate their cuticular microbiomes are very similar despite their geographic separation (South America and Africa, respectively). Notably, antibiotic-producing actinomycete bacteria represent a tiny fraction of the cuticular microbiomes of both *Allomerus* and *Tetraponera* spp. and instead they are dominated by γ-proteobacteria *Erwinia* and *Serratia* spp. Both these phyla are known to contain antibiotic-producing species which might therefore play a protective role in these ant–plant systems.

## Introduction

Insect–bacterial symbioses are widespread in the environment (Moran 2006) and antibiotic-producing bacterial symbionts are often used to protect the host and/or their resources (Seipke et al. 2012a). In recent years, these symbioses have been explored as a potential source of new antibiotics as bacteria that have coevolved with their hosts might have evolved unique antibiotic-biosynthetic pathways (Poulsen 2010). Many insects (ants, termites, gall midges, and beetles) have evolved fungiculture and live in symbiosis with their fungal cultivar. In attine ants, it has been shown that mixed communities of antibiotic-producing bacteria (Phylum Actinobacteria) protect the ants and possibly their fungal cultivar against infection (Currie et al. 1999; Haeder et al. 2009; Sen et al. 2009; Barke et al. 2010; Schoenian et al. 2011; Mattoso et al. 2012).

Fungiculture has also been suggested to be widespread in plant–ants, which live in symbiosis with a host plant (Defossez et al. 2009). Recently discovered examples include *Allomerus* and *Tetraponera* species, which both use a single type of fungus, but for very different purposes. *Allomerus* spp. live in symbiosis with the Amazonian plant, *Hirtella physophora*, and cultivate a sooty mold (order Chaetothyriales) to use as a building material to carry out their unique mechanism of ambush hunting (Heil and McKey 2003; DeJean et al. 2005). These ants use their fungus to build a galleried structure along the stems and branches of their host plants in which they hide and await insect prey (DeJean et al. 2005; Ruiz-Gonzalez et al. 2010). The fungus may also act as a chemoattractant to prey insects (DeJean et al. 2005; Ruiz-Gonzalez et al. 2010). *Tetraponera* spp. live in symbiosis with the African plant, *Acacia drepanolobium* (swollen thorn acacia), which dominates the vegetation of the Kenyan Laikipia District (Young et al. 1997). The acacia defends itself against large- and medium-sized herbivores by a combination of long hollow thorns and by hosting four different ant species of the genera *Tetraponera* and *Crematogaster* (Young et al. 1997; Palmer et al. 2008). These ants have been observed cultivating a fungus (genus *Chaetomium*) inside their domatia and recent evidence suggests that they cultivate this fungus as food (Defossez et al. 2009). Remarkably, *Tetraponera* spp. also host nitrogen-fixing endosymbiotic bacteria, related to those found in the root nodules of legumes, which are proposed to fix nitrogen for their hosts (Van Borm et al. 2002).

The purpose of our study was to investigate the bacterial communities on the cuticles of fungus-growing *Allomerus* and *Tetraponera* ants and in particular to discover whether antibiotic-producing actinobacteria predominate in these communities as they do in attine ants. We previously reported that antibiotic-producing actinobacteria can be isolated from *Allomerus* spp. using culture-dependent techniques. However, selective isolation of these actinomycetes gave no indication as to their abundance on *Allomerus* workers or in their domatia and offered no evidence of a meaningful or specific interaction (Seipke et al. 2012c). In this study, we use culture-independent 16S rDNA 454-pyrosequencing to examine the bacterial communities associated with *Allomerus* and *Tetraponera* worker ants. We report that the cuticular microbiomes of *Allomerus* and *Tetraponera* spp. are dominated by Proteobacteria, but share a very similar distribution of bacterial phyla despite their geographic separation. At the genus level, the proteobacterial genera *Erwinia* and *Serratia* are dominant and both are known to include antibiotic-producing species. A single antibiotic-producing actinomycete (*Streptomyces* spp.) was found in the *Allomerus* samples and none were found associated with *Tetraponera* ants, suggesting that actinomycetes do not play an important role in this symbiosis. We conclude that if these plant–ants are using antibiotics to protect themselves or their fungal cultivars then they are most likely supplied by *Erwinia* or *Serratia* species.

## Materials and Methods

### Sample collection

*Allomerus* samples were collected in French Guiana from three inland sites, Basevie, Area 9, and Area 24, which all are close to the field station at Barrage de Petit Saut. *Hirtella*-hosted *A. decemarticulatus* colonies (HPAD) were sampled as well as *Cordia*-hosted *A. octoarticulatus* colonies (CNAO) and *Hirtella*-hosted *A. octoarticulatus* (HPAO) colonies. Five worker ants from each of the HPAD, CNAO, and HPAO samples were pooled and stored in 1 mL of 20% glycerol at −20°C, until processing. *Allomerus decemarticulatus* and *A. octoarticulatus* ants were distinguished morphologically by counting the number of antennal segments of representative worker ants under a stereomicroscope, 10 for *A. decemarticulatus* and eight for *A. octoarticulatus*. *Tetraponera penzigi* workers were provided by Naomi E. Pierce (Harvard, U.S.A.) and were sampled from Kitengela (S1^o^23′526′′ E36^o^49′108′′) and Ngong Hills (S1^o^26′946′′ E36^o^38′358′′) from southern Kenya (near Nairobi), as well as Mpala Road (N0^o^32′ E36^o^42′) from central Kenya (Laikipia district). An average of five worker ants were collected from each colony and were preserved in 1 mL of 20% glycerol.

### Isolation of bacterial DNA, PCR, sequencing, and analysis

Bacteria present on worker ants stored in 20% glycerol were removed by vortexing and were subsequently trapped by passing the glycerol solution through a 0.45-μm filter. Total DNA was extracted from the filters using phenol–chloroform extraction followed by ethanol precipitation. The DNA was used as a template for PCR with the universal primers Gray28F 5′-GAGTTTGATCNTGGCTCAG and Gray519R 5′-GTNTTACNGCGGCKGCTG, which target variable regions V1–V3 in the 16S gene (Ishak et al. 2011b). A multiplex identification tag (11 nucelotides) was ligated to the 5′ and 3′ ends of 16S amplicons prior to 454-pyrosequencing (1/6 plate per sample) with a GS FLX sequencer (Roche, Hertfordshire, UK) using the GS FLX Titanium series chemistry kit at The Genome Analysis Centre (Norwich, U.K.). Raw data were deposited in the European Nucleotide Archive under accessions ERX177635 and ERX177636 for *Tetraponera* and *Allomerus* samples, respectively. The resulting 454 sequence reads were processed and analyzed using the software package Quantitative Insights Into Microbial Ecology v1.4.0 (http://qiime.org/index.html, Caporaso et al. 2010b) and the software packages contained within using the default parameters for each step unless otherwise specified. Reads that lacked the primer or possessed more than one primer mismatch, were too short (<200 nt), or contained a homopolymer run exceeding six nucleotides were removed. The remaining sequences were grouped into samples according to their 11-mer identifier and subsequently denoised using the denoise_wrapper.py script in QIIME (Reeder and Knight 2010). Denoised sequence reads were binned into operational taxonomic units (OTUs) using UCLUST (Edgar 2010) with shared identity threshold of 97%. Chimeric OTUs were identified by ChimeraSlayer (Haas et al. 2011) and discarded. One representative sequence from each OTU was aligned to the Greengenes core reference alignment (DeSantis et al. 2006) with PyNAST with a minimum percent identity of 75% (Caporaso et al. 2010a). Taxonomy was assigned using the Ribosomal Database Project (RDP) classifier 2.2 (Wang et al. 2007) with a minimum support threshold of 80%. Sequences belonging to OTUs designated as unclassified or that could not be assigned taxonomy beyond the domain Bacteria were removed and assumed to be derived from nonbacterial 16S sequences.

## Results and Discussion

*Allomerus* samples were collected in French Guiana and *Tetraponera* samples were collected in Kenya (see Materials and Methods). Bacteria were washed off from ant cuticles using sterile 20% glycerol and bacterial DNA was extracted and used as template for PCR with universal primers Gray28F and Gray519R (Materials and Methods). The 16S amplicons that resulted were 454-pyrosequenced with a GS FLX sequencer (Roche) using the GS FLX Titanium series chemistry kit. Sequence data were quality filtered and taxonomy was assigned using the Quantitative Insights Into Microbial Ecology (QIIME) software package (Caporaso et al. 2010b). In total, 16,427 pyrotags (*Allomerus*) and 8388 pyrotags (*Tetraponera*) passed quality filtering and were assigned taxonomy.

**Figure 1:**
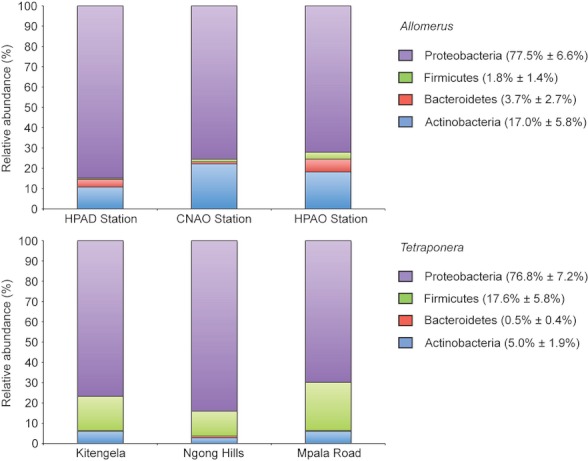
Relative pyrotag abundance of phyla in cuticular microbiomes of *Allomerus* and *Tetraponera* ants. The cuticles of both ant genera are dominated by Proteobacteria. We also report the average relative abundance ± the standard error for each phyla observed. For the *Tetraponera* graph, Cyanobacteria and candidate phylum TM7 are not shown but are present in average abundances of <0.01%.

The relative abundance of bacteria phyla on cuticles of *Allomerus* and *Tetraponera* ants is shown in Figure 1. Proteobacteria accounted for >75% of all pyrotags for both *Allomerus* and *Tetraponera* spp. Roughly 17% of pyrotags were classified as Actinobacteria (*Allomerus*) and Firmicutes (*Tetraponera*), while Firmicutes (1.8% of pyrotags) and Bacteroidetes (0.5% of pyrotags) were the least abundant phyla in *Allomerus* and *Tetraponera* cuticular microbiomes, respectively.

**Figure 2:**
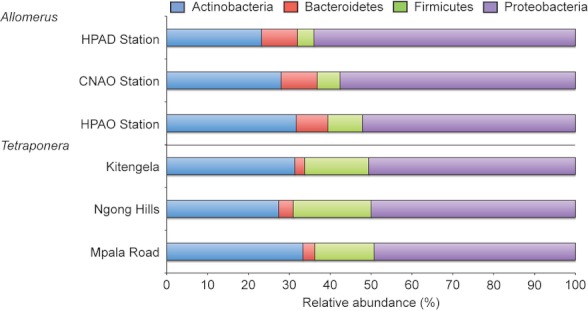
Relative operational taxonomic unit (OTU) richness of bacterial phyla in cuticular microbiomes of *Allomerus* and *Tetraponera* ants. For *Allomerus*, two Proteobacteria OTUs that could not be assigned to a class were omitted from the analysis.

Pyrotags were binned into OTUs sharing ≥97% identity and assigned taxonomy. The bacterial diversity at the phylum level is summarized in Table 1 and the relative abundance of these phyla are graphically depicted in Figure 2. Proteobacteria accounted for ∼58% of OTUs for *Allomerus*, and ∼50% of OTUs for *Tetraponera* on average. For *Allomerus*, OTU richness for the remaining phyla correlated perfectly to the pyrotag abundance displayed in Figure 1. However, for *Tetraponera*, despite accounting for only ∼5% of pyrotags, Actinobacteria possessed greater bacterial diversity (an average of 20 OTUs) compared with Firmicutes, which accounted for ∼17% of pyrotags, but only consisted of 11.7 OTUs on average.

**Table 1:** Richness of operational taxonomic units (OTUs) for *Allomerus* and *Tetraponera* ant cuticular microbiomes

Phylum	*Allomerus*					*Tetraponera*				
	
	HPAD Station	CNAO Station	HPAO Station	Avg	StErr	Kitengela	Ngong Hills	Mpala Road	Avg	StErr
Actinobacteria	29	35	45	36.3	8.1	26	23	23	20.0	2.0
Bacteroidetes	11	11	11	11.0	0.0	2	3	2	2.3	0.6
Firmicutes	5	7	12	8.0	3.6	13	16	10	11.7	2.5
Proteobacteria	80	72	74	75.3	4.2	42	42	34	39.0	4.4
Total	125	125	142	130.7	9.8	83	84	69	78.7	8.4

OTUs comprise pyrotags sharing ≥97% nucleotide identity. HPAD, *Hirtella*-hosted *A. decemarticulatus*; CNAO, *Cordia*-hosted *A. octoarticulatus*; HPAO, *Hirtella*-hosted *A. octoarticulatus*; Avg, average; StErr, standard error.

Strikingly, of the OTUs classified as Actinobacteria, *Allomerus* ants possessed only a single OTU (0.06% relative abundance of pyrotags for the CNAO sample) corresponding to a known antibiotic-producing actinomycete genus (*Streptomyces*) and the *Tetraponera* sample did not possess any OTUs classified as antibiotic-producing Actinobacteria. This starkly contrasts with the cuticular microbiome of attine ants, which is dominated by antibiotic-producing actinomycetes *Pseudonocardia* and *Amycolatopsis* spp. (Sen et al. 2009; Ishak et al. 2011a; Anderson et al., 2011), and is also dissimilar from the microbiome of fire ant workers, which are dominated by *Spiroplasma* and *Nocardia* spp. (Ishak et al. 2011b).

**Figure 3:**
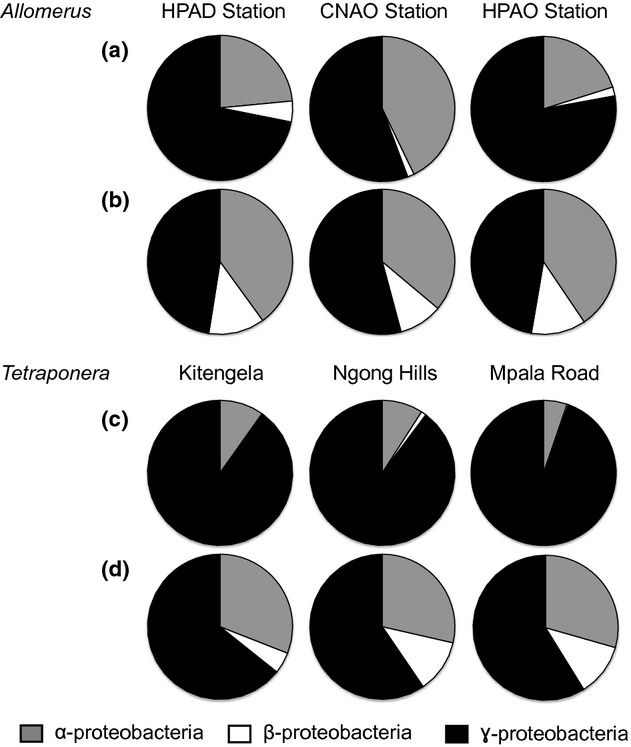
The diversity of Proteobacteria within *Allomerus* and *Tetraponera* cuticular microbiomes. (A and C) Relative abundance of pyrotags from proteobacterial classes; (B and D) Relative abundance of operational taxonomic units (OTUs) (97% identity) from proteobacterial classes. The distribution and average relative abundance of Proteobacteria pyrotags and OTUs is enumerated in Table S1.

As Proteobacteria was the dominant phyla identified both in terms of pyrotag abundance and OTU richness, we analyzed the diversity of Proteobacteria at the level of class (Fig. 3). γ-Proteobacteria was the most abundant class, accounting for 68% and 91% of pyrotags, and 49% and 61% of OTU richness for *Allomerus* and *Tetraponera* ants, respectively (Table S1). The decrease in relative abundance of γ-proteobacteria pyrotags versus OTU richness suggests that a few taxa account for the majority of Proteobacteria pyrotags. In order to further analyze this possibility, we tabulated OTUs with a pyrotag relative abundance ≥1% (Table 2). Bacterial types at greater than 84% abundance in the microbial community associated with *Allomerus* ants is represented by 36 species, with a single *Erwinia* species (29.2%), two *Ochrobactrum* species (10.6%), and a single *Serratia* species (10.2%) comprising the most abundant taxa. Likewise, greater than 84% of the microbial community associated with the cuticles of *Tetraponera* ants is represented by just 15 genera, with two *Serratia* species being the most abundant (53.6%). Rarefaction plots using 97% shared identity (i.e., species-level classification) (Fig. S1) indicated that our sampling captured the majority of the bacterial diversity at this taxonomic level associated with cuticles of *Allomerus* and *Tetraponera* ants, though it is likely we undersampled rare bacterial genera.

**Table 2:** Genera of OTUs associated with *Allomerus* and *Tetraponera* ant cuticles

*Allomerus*	Phylum or class	HPAD Station	CNAO Station	HPAO Station	Avg	StErr
Quality-filtered pyrotags		5312	5661	5454		
Genus						
*Actinomycetales*^1^	Actinobacteria	0.6	–	1.1	0.6	0.6
*Actinomycetales*^1^	Actinobacteria	–	1.1	1.0	0.7	0.6
*Microbacterium*	Actinobacteria	0.3	8.9	–	3.1	5.1
*Actinomycetales*^1^	Actinobacteria	0.1	1.1	0.2	0.5	0.6
*Microbacterium*	Actinobacteria	6.3	1.2	8.0	5.2	3.5
*Brevibacterium*	Actinobacteria	–	3.4	0.6	1.3	1.8
*Actinomycetales*^1^	Actinobacteria	0.9	2.0	1.5	1.4	0.5
*Sphingobacterium*	Bacteroidetes	0.3	0.1	1.2	0.5	0.6
*Sphingobacterium*	Bacteroidetes	–	–	2.6	0.9	1.5
*Sphingobacterium*	Bacteroidetes	1.6	0.4	0.6	0.9	0.7
*Bacillus*	Firmicutes	0.5	0.1	1.9	0.8	1.0
*Staphylococcus*	Firmicutes	<0.1	1.1	0.4	0.5	0.6
*Bosea*	α-proteobacteria	0.8	0.1	1.8	0.9	0.8
*Devosia*	α-proteobacteria	–	<0.1	1.1	0.4	0.7
*Rhizobium*	α-proteobacteria	1.3	0.2	0.2	0.6	0.7
*Phyllobacteriaceae*^2^	α-proteobacteria	1.1	<0.1	–	0.4	0.6
*Bradyrhizobiaceae*^2^	α-proteobacteria	0.8	0.1	1.3	0.7	0.6
*Rhizobiales*^1^	α-proteobacteria	2.7	3.5	2.5	2.9	0.5
*Ochrobactrum*	α-proteobacteria	2.6	10.4	1.3	4.8	4.9
*Ochrobactrum*	α-proteobacteria	4.6	12.4	0.2	5.8	6.2
*Bosea*	α-proteobacteria	1.7	2.4	1.3	1.8	0.6
*Rhizobium*	α-proteobacteria	0.9	<0.1	1.5	0.8	0.7
*Bradyrhizobiaceae*^2^	α-proteobacteria	0.7	1.6	0.3	0.9	0.6
*Comamonas*	β-proteobacteria	1.4	–	–	0.5	0.8
*Shinella*	β-proteobacteria	0.3	0.3	1.1	0.6	0.4
*Shinella*	β-proteobacteria	1.0	0.1	0.1	0.4	0.5
*Enterobacteriaceae*^2^	γ-proteobacteria	0.8	0.3	1.6	0.9	0.7
*Stenotrophomonas*	γ-proteobacteria	<0.1	1.0	0.6	0.5	0.5
*Serratia*	γ-proteobacteria	12.5	15.6	1.4	9.8	7.5
*Stenotrophomonas*	γ-proteobacteria	1.9	4.4	0.9	2.4	1.8
*Enterobacteriaceae*^2^	γ-proteobacteria	0.1	4.6	<0.1	1.6	2.6
*Enterobacteriaceae*^2^	γ-proteobacteria	1.1	0.5	1.6	1.1	0.5
*Enterobacteriaceae*^2^	γ-proteobacteria	<0.1	0.5	1.1	0.5	0.5
*Erwinia*	γ-proteobacteria	38.6	5.3	43.7	29.2	20.8
*Enterobacteriaceae*^2^	γ-proteobacteria	1.1	1.1	1.9	1.4	0.5
*Enterobacter*	γ-proteobacteria	0.4	3.7	0.4	1.5	1.9
Total		86.9	87.5	84.8		

*Tetraponera*		Kitengela	Ngong Hills	Mpala Road		
Quality-filtered pyrotags		2709	2601	3078		
Genus						
*Curtobacterium*	Actinobacteria	1.0	0.3	0.3	0.5	0.4
*Microbacteriaceae*^2^	Actinobacteria	1.6	0.8	1.8	1.4	0.5
*Actinomycetospora*	Actinobacteria	<0.1	0.1	1.4	0.5	0.8
*Staphylococcus*	Firmicutes	–	0.5	19.8	6.7	11.3
*Geobacillus*	Firmicutes	10.2	8.2	2.8	7.1	3.8
*Weissella*	Firmicutes	4.4	0.9	–	1.8	2.3
*Rhizobiales*^1^	α-proteobacteria	1.6	0.8	0.4	0.9	0.6
*Sphingomonadaceae*^2^	α-proteobacteria	0.7	5.3	–	2.0	2.9
*Brevundimonas*	α-proteobacteria	1.3	0.1	0.4	0.6	0.6
*Sphingobium*	α-proteobacteria	<0.1	–	1.1	0.4	0.6
*Serratia*	γ-proteobacteria	1.9	3.5	3.3	2.9	0.9
*Serratia*	γ-proteobacteria	27.6	64.8	59.6	50.7	20.2
*Enterobacteriaceae*^2^	γ-proteobacteria	2.7	–	–	0.9	1.5
*Enterobacteriaceae*^2^	γ-proteobacteria	30.7	–	<0.1	10.2	17.7
*Halomonas*	γ-proteobacteria	0.8	1.0	0.2	0.7	0.4
Total		84.4	86.2	91.1		

Only OTUs comprising ≥1% relative pyrotag abundance are shown. Also shown is the average percent abundance (Avg) and standard error (StErr) of taxa. Taxonomy at the level of class is shown for Proteobacteria. OTU, operational taxonomic unit; HPAD, *Hirtella*-hosted *A. decemarticulatus*; CNAO, *Cordia*-hosted *A. octoarticulatus*; HPAO, *Hirtella*-hosted *A. octoarticulatus*.

The ^1^order or ^2^family is reported in instances when a genus-level classification could not be made.

The high abundance of *Erwinia* spp. in the *Allomerus* samples may not be surprising, because this genus of bacteria are abundant epiphytic phytopathogens that cause disease on crop plants and crop trees (Starr and Chatterjee 1972). The γ-proteobacteria *Erwinia* species associated with *Allomerus* ants is possibly carryover from the close association with their plant host. The α-proteobacterium *Ochrobactrum* and γ-proteobacteria *Serratia* spp. are well-known insect symbionts (both harmful and beneficial) and more than 70 species of insects are susceptible to infections by *Serratia* spp. (Bucher 1963; Grimont and Grimont 1978). One highly coevolved species (Candidatus *Serratia symbiotica*) is an important endosymbiont of the aphid, *Cinara cedri* (Lamelas et al. 2008). It is notable that *Tetraponera* ant samples from three separate sites were dominated by *Serratia* species with an abundance as high as 64.8% in the Ngong Hills sample. In addition to their roles as plant and insect pathogens or symbionts, *Erwinia* and *Serratia* spp. are also antibiotic producers (Wilf and Salmond 2012), which suggests that *Allomerus* and *Tetraponera* ants may use these taxa for protection. Certainly, the dominance of *Serratia* species on the *Tetraponera* worker ants is reminiscent of the levels of abundance of actinomycetes in attine ants (Sen et al. 2009; Anderson et al. 2011; Ishak et al. 2011a).

Given that we previously isolated six actinobacterial species from *Allomerus* ants (one *Amycolatopsis* spp. and five *Streptomyces* spp.) and four of the isolates produced antifungal compounds (Seipke et al. 2012c), it is somewhat surprising that antibiotic-producing actinobacteria are not abundant on *Allomerus* ants. Additionally, even though our sampling suggested the absence of antibiotic-producing actinobacteria within the cuticular microbiome of *Tetraponera* ants, we could readily isolate antibiotic-producing actinobacteria from the ants used in this study, including three *Streptomyces* spp. and three *Saccharopolyspora* spp, five of which have antifungal activity (Table S2). In fact, this highlights the caution with which culture-dependent studies should be treated as our own approach was biased toward the selective isolation of actinomycetes, which have a “hairy” colony morphology due to their filamentous growth and are easy to identify by eye on agar plates. The culture-independent data suggests that filamentous actinomycete bacteria have a rare abundance on *Allomerus* and *Tetraponera* spp. as most of the species observed in culturing studies were not detected by deep sequencing the same samples (Materials and Methods). As noted above, however, it is likely we under-sampled rare bacterial genera.

Our direct sequencing approach suggests that it is unlikely that *Allomerus* and *Tetraponera* species use actinomycetes for protection and instead suggest that antibiotic-producing Proteobacteria might perform this role. This would make sense as these plant–ants rarely, if ever, leave their host plants and do not come into contact with the soil where actinomycete species commonly reside. Alternatively, they will come into frequent contact with *Erwinia* and *Serratia* spp. because they are common symbionts of plants and insects and so these bacteria may have colonized or been recruited to the plant–ant cuticles. It is also possible that the ants do not need protection from antibiotic-producing bacteria, for example, they are less exposed to pathogens because they do not leave their host plants and do not bring exogenous material (e.g., leaf matter) into the nest; or they may have evolved efficient grooming techniques that are sufficient to limit spread of pathogens in the nest and they may produce their own antimicrobial compounds and these are sufficient to protect against infection.

## Conclusions

Our study supports the hypothesis that plant–ants like *Allomerus* and *Tetraponera* are colonized by very different bacterial phyla compared with soil-dwelling ants like the attines. It seems likely that attine ants recruited antibiotic-producing actinomycete bacteria from the soil and evolved a symbiosis in which they use these bacteria for protection. We propose that a similar scenario might be true for plant–ants but in their case they used antibiotic-producing bacteria commonly found on their host plants or prey insects, notably *Erwinia* and *Serratia* spp. Certainly it is striking that worker ants of both ant–plant systems are dominated by the same bacterial phyla and future work will be aimed at understanding the role that these bacterial species play in ant–plant–fungal symbioses.

## Supporting Information

**Figure S1.** Rarefaction curves (97% shared identity) for *Allomerus* (HPAO Station, HPAD Station, CNAO Station) and *Tetraponera* (Ngong Hills, Kitengela, Mpala Road) ant samples.

**Table S1.** Distribution of Proteobacteria within cuticular microbiomes *Allomerus* and *Tetraponera* ants.

**Table S2.** 16S identification of the antibiotic-producing Actinobacteria genera from *Tetraponera* ants.
