# Supplementary figures and images for: Analysis of the bacterial communities associated with two ant–plant symbioses
Source: Microbiologyopen. 2013 Feb 17;2(2):276–83. doi: 10.1002/mbo3.73 (PMC3633351; doi:10.1002/mbo3.73)

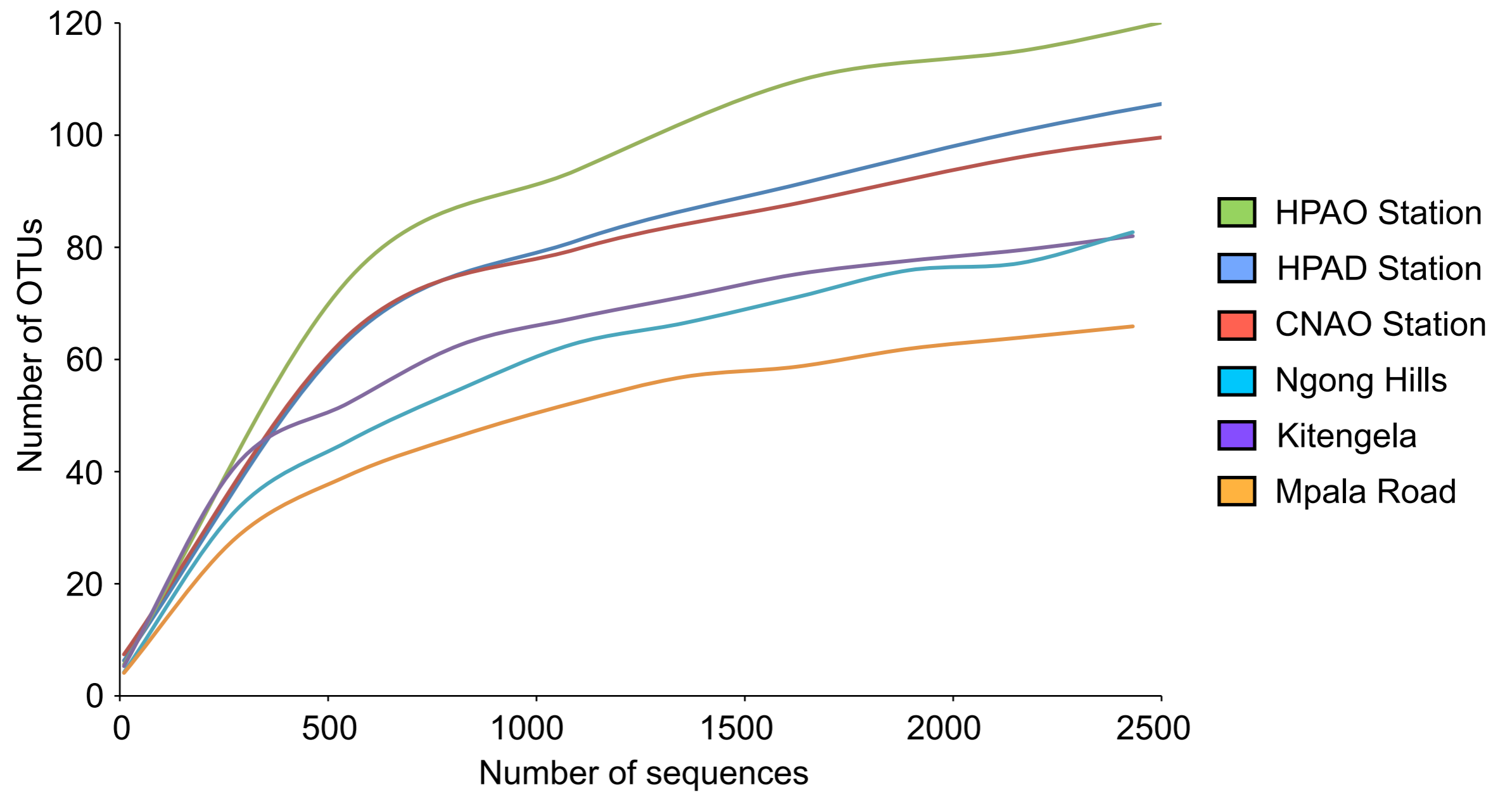

Supplement: Supplementary file 1 [file mbo30002-0276-SD1.pdf]
