# Supplementary material for: Analysis of the bacterial communities associated with two ant–plant symbioses
Source: Microbiologyopen. 2013 Feb 17;2(2):276–83. doi: 10.1002/mbo3.73 (PMC3633351; doi:10.1002/mbo3.73)
Supplement: Supplementary file 3 [file mbo30002-0276-SD2.pdf]

	Allomerus		*Tetraponera*	
Class	% of pyrotags	OTUs	% of pyrotags	OTUs
α-proteobacteria	28.7	38.9	8.2	29.7
β-proteobacteria	2.8	11.5	0.6	9.3
γ-proteobacteria	68.5	49.6	91.2	61.0

Table S1. Distribution of Proteobacteria within cuticular microbiomes *Allomerus* and *Tetraponera* ants. Values represent the average relative abundance of either pyrotags or OTUs classified as belonging to the phylum Proteobacteria.
