# Supplementary material for: Analysis of the bacterial communities associated with two ant–plant symbioses
Source: Microbiologyopen. 2013 Feb 17;2(2):276–83. doi: 10.1002/mbo3.73 (PMC3633351; doi:10.1002/mbo3.73)
Supplement: Supplementary file 5 [file mbo30002-0276-SD3.pdf]

Strain	Source location	Accession	Genus	Antifungal activity	Closest relative (accession, % identity)
T1	Kitengela	JX305999	*Streptomyces*	Y	*Streptomyces* sp. OA18 (JN942133, 100)
T2	Ngong Hills	JX306000	*Streptomyces*	Y	*Streptomyces* sp. FXJ7.385 (JF346529, 100)
T3	Ngong Hills	JX306001	*Saccharopolyspora*	Y	*Saccharopolyspora* sp. 8-15 (HQ622522, 99)
T4	Ngong Hills	JX306002	*Streptomyces*	N	*Streptomyces* sp. CPE1 (JN936839, 100)
T7	Kitengela	JX306003	*Saccharopolyspora*	Y	*Saccharopolyspora* sp. JAJ12 (JN859001, 99)
T21	Ngong Hills	JX306004	*Saccharopolyspora*	Y	*Saccharopolyspora* sp. JAJ12 (JN859001, 99)

Table S2. 16S identification of the antibiotic-producing actinobacteria genera from *Tetraponera* ants. Genbank accession numbers are given for the 16S sequences generated from this study and the closest relative and % identity is indicated. Bacteria were isolated and assessed for antifungal activity against *Candida albicans* as previously described (Seipke *et al.*, 2012b).
